# Caries and Necrosis of Alveoli of Right Superior Maxilla and Chronic Inflammation of Antrum, Due to Excessive Salivary Concretions and a Neglected Pulpless Molar

**Published:** 1886-04

**Authors:** B. Merrill Hopkinson

**Affiliations:** Baltimore.


ARTICLE II.

### CARIES AND NECROSIS OF ALVEOLI OF RIGHT SUPERIOR MAXILLA AND CHRONIC INFLAMMATION OF ANTRUM, DUE TO EXCESSIVE SALIVARY CONCRETIONS AND A NEGLECTED PULPLESS MOLAR.

BY B. MERRILL HOPKINSON, D. D. S., M. D., OF BALTIMORE.

I have had in the course of my practice during the past few years, what would seem to be, to judge from the experience of others, a large number of cases of diseases of the maxillary sinus. With few exceptions, the disease has been devoid of active symptoms, at the time I found it, the inflammation being of a chronic nature and showing itself either in the nasal cavity, or in the throat, rather than in the oral cavity and in connection with the dental organs. I have generally discovered these troubles in operations upon the teeth, either in extractions of teeth or roots, or in the treatment of these organs when in a pulpless state. The finding of incrustations of salivary calculus upon the teeth is of daily, almost hourly occurrence, and it is most surprising how great the accumulation may be, without ordinarily producing serious injury. The appended case, of which I give a record below, is one of those rare instances where caries and necrosis of the alveoli, together with antrum disease and its attendant evils, may be caused by great accumulations of calculus, complicated with a neglected pulpless tooth. I have never seen a similar case recorded, and I think it most interesting for several reasons. I wish it were possible for medical men in general practice to be a little better acquainted with the diseases treated by oral specialists. It would indeed be a good thing for suffering mankind, and I am sure that thousands of patients would yearly save their valuable organs of mastication, and also be saved a great amount of unnecessary pain if such were the case. I have my attention called to this defect of education, as well as of study, time and again; and in the case below the young man had been variously treated, without lasting benefit of course. for general debility, malaria, &c,, *ad infin*, before I was so fortunate as to discover the true cause of his trouble when he applied to me for oral services. The usual symptoms of antrum disease were absent; there did not seem to be any special indication, from an oral inspection, that would lead one to anticipate the finding of a bone lesion; indeed it was an obscure case, and yet the simple removal of salivary incrustations, as well as a tooth broken down with caries and containing a dead pulp, made the diagnosis clear, and placed the man in a condition to recover his health and strength. I would most respectfully suggest that general practitioners pay more attention to the condition of the mouths of their patients, and advise a more frequent calling upon the dental surgeon; for I am fully convinced from experience and observation that nine-tenths of those suffering from general maladies would be greatly benefitted by such visits, and the services of each would aid the other.

Mr. S., office clerk, bilious-nervous temperament, age 21, consulted me June 18, 1885, concerning his oral cavity, which was sadly in need of treatment. Notwithstanding the fact that he is a young man of good education and surroundings, he had neglected his mouth in a shameful manner, the result being vitiated secretions, most imperfect mastication and consequent indigestion, and a general condition of ill-health. He complained of pain of a general character, and it was difficult to localize any particular lesion, on account of the wretched condition of a number of teeth, and the fact of there being no special soreness or tumefaction present; but, from symptoms as described, and having found pus mixed with the nasal secretions, and detected a bad odor not due to carious teeth, I diagnosed, together with dental troubles, disease of a deeper seated nature than that of simple caries of these organs, and began to look elsewhere for the principal seat of his difficulty. After careful examination into his history I eliminated specific disease. He was very anaemic, complained of weakness, anorexia, and inability to apply himself steadily at his avocation; was habitually constipated; thought, as most of the sick laity do in these days, that he was suffering from aggravated Bright's, and was generally miserable and depressed. A prominent symptom was profuse and obstinate epistaxis principally from the right side, that nostril being almost continually occluded, save at these periods. I immediately proceeded, as the first step in local treatment, as well as an aid to diagnosis, to condemn and advise the extraction of seven teeth, and having the concurrence of my patient commenced operating. I removed five teeth with the patient in a conscious state, and without the aid even of a local obtundent, but as soon as I arrived in turn at the superior right second molar, he suddenly refused most positively to have me go on, saying that he had an indefinable fear of having that and the adjoining tooth removed, unles he was first rendered unconscious. There were no external evidences of disease, save enormous incrustations of salivary calculus, and unhealthy appearance of the gums, due to that cause; but, persuasion being useless, I allowed him to inhale a suitable quantity of nitrogen monoxide, and, first having removed a sufficient amount of the calculus, applied the forceps to the second molar and with surprising ease removed it, brought with it an inch of the alveolar border, loosened the third molar, and left the gums hanging like two curtains uninjured. I immediately removed the third molar, which brought with it a portion of diseased alveolus, leaving the gums as before, there appearing to be no connection between them and the bony parts which came away.

Immediately following extraction came a profuse flow of blood mixed freely with offensive pus, giving unmistakable evidences of necrosis. His reaction, as is the rule when $N_2O$ is administered, was rapid, complete and satisfactory. I explained to him the condition of affairs and commenced a careful examination with a view of discovering the extent of the disease. I found I had removed the alveolar border entire, down to the body of the bone to the extent of about one and one-half inches. I proceeded to remove all further discoverable portions of carious bone, carefully washed the parts with a carbolized solution, and in continuing my examination found that a probe could be easily passed through quite a large opening into the antrum and injections through the opening found their way most readily into the nasal cavity. I found here then the seat and principal cause of all his trouble. This state of things had evidently existed for some time and he was quite unaware of any disease of the parts, anything more than a general uneasiness which he attributed to a carious tooth. The removal of the calculus and tooth removed, of course, the cause of the disease, made a diagnosis sure, and laid the foundation for permanent general improvement. Everything seemed favorable for successful local treatment, the tendency to haemorrhage seemed *nil*, there being very little bleeding since the first gush after removal of the teeth, and after thorough injections with a tepid carbolized solution, two per cent., a suitable dressing of absorbent cotton, saturated with the solution to keep the wound patulous, permit drainage and prevent sepsis, which dressing the pendent gums euabled me to apply satisfactorily, I dismissed him for the day, and enjoined him to remain as quiet as possible and to return next day. Before leaving I prescribed a pill to be taken daily, or on each alternate day as required, at bed time, containing res. podophyl gr. 1-5, aloes aq. ext. gr. ⅓, to regulate his bowels, told him to bring me a sample of urine for examination, which I afterwards found to be normal, and this fact enabled me to aid him in dismissing from his mind the awful bugbear of morbus Brightii. On the following day, I found that he had disobeyed my injunction to keep quiet, and had managed to get my dressing displaced, and in so doing had caused a slight haemorrhage which alarmed him, and he applied immediately for professional services in the locality in which he happened to be. My worthy friend and brother succeeded in stopping the haemorrhage, and also, in doing just that which I desired most to avoid, viz: using a solution of an iron salt, and plugging up the cavity so tight that proper drainage was completely arrested, and my young man presented himself in a sorry plight with a very swollen face. I removed the great mass of cotton causing hæmorrhage again, but it was easily overcome by tepid injections, thoroughly injected the antrum and other diseased parts with solution as before, renewed the dressing of the previous day, and also my injunction as to quiet. I am of the opinion that the use of Monsell's solution as a stypic is seldom required in oral, or indeed in general practice, and it should be avoided when possible, the formation of an insoluble clot followed by sloughing naturally causing a retardation of the healing process. The same local treatment was continued for nine days with very satisfactory results and the progress towards recovery was remarkably rapid. The pills operated very kindly and after four days it was only necessary to repeat them on each alternate day. I prescribed for him tr. ferri. muriat. gtt. x et quiniae sulph. grs. ij to be taken thrice daily; at the end of the nine days I omitted the dressing, previous symptoms had disappeared, and he was in condition to have seventeen fillings inserted in his remaining teeth, which operations I commenced shortly after. On October 29, or a little over four months from first operations, I

inserted for him a partial superior denture, filling all vacancies, which he is wearing with much comfort, and is now in full possession and enjoyment of good health. The diseased tooth I found upon examination to contain a mummified dead pulp, the roots wers greatly exostosed, in fact, were three times their normal size, judging from the size of roots of his other teeth, were firmly adherent to their alveoli, and the palatine root had evidently penetrated the antrum. There were no signs of alveolar abscess. This case demonstrates clearly two things; first, that excessive salivary concretions may do a great deal of harm, though such cases are most rare, and that when calculus is present upon the teeth it should invariably be removed. Secondly, that it is dangerous to leave a pulpless tooth in the dental arch *unattended to.* I will say in conclusion that the latter class of teeth, of which the dentist finds a great many, may *by proper treatment* be made wholly innocuous, and put in a condition to perform their proper functions with perfect comfort and satisfaction for an unlimited number of years. I speak decisively and authoritatively on the subject of pulpless teeth, from the fact that I have read during the past year extracts from papers of a prominent physician in which he condems what *he* calls "*dead teeth*" in a wholesale and finished manner. Such condemnation is not due to ignorance, but to lack of a critical examination of the subject. Pulpless teeth are not *dead*, they have only lost their inner life, that of the pulp, and still receive adequate nourishment through the periosteum to preserve their vitality, in part, indefinitely. If they were *dead*, the condemnation of my learned brother would be just, and if not removed by the specialist, nature herself would not tolerate them but would cast them off as bodies, foreign to the animal economy.—*Md. Med. Journal.*

